# Sleep and executive functions in older adults: A systematic review

**DOI:** 10.1590/S1980-5764-2016DN1003004

**Published:** 2016

**Authors:** Francisco Wilson Nogueira Holanda, Katie Moraes de Almondes

**Affiliations:** 1 Master's Student on the Postgraduate Program in Psychology, Federal University of Rio Grande do Norte.; 2 Associate Professor at the Department of Psychology and on the Postgraduate Program in Psychology, Federal University of Rio Grande do Norte, Natal RN, Brazil.

**Keywords:** executive functions, sleep, older adults, prefrontal cortex

## Abstract

**Introduction:**

A recent increase in studies suggests a role of age-related sleep changes in executive functions (EF). However, this relationship remains unclear and mixed results have emerged.

**Objective:**

To investigate how age-related sleep changes may play an important role in the extent to which healthy older adults exhibit decline in EF.

**Methods:**

A systematic strategy was employed to identify the available literature on age-related sleep changes and EF. Results: Of the 465 studies identified, 26 were included.

**Results:**

suggest that multiple sleep parameters differ in the way they benefit or impair EF. Parameters such as greater wake after sleep onset and lower sleep efficiency, in addition to circadian fragmentation of sleep, showed more consistent results and are potentially correlated with worsening in EF measures. However, other results seem inconclusive.

**Conclusion:**

These findings were discussed based on the prefrontal circuitry vulnerability model, in which sleep has been identified as a beneficial factor for prefrontal cortex functioning and hence for EF, which relies mostly on this brain area and its related networks.

## RESUMO

**Introdução:**

Um aumento recente de estudos sugere um papel das mudanças do sono relacionadas ao envelhecimento nas funções executivas (FE). Entretanto, essa relação ainda não é clara e resultados mistos têm emergido.

**Objetivo:**

Investigar como as mudanças do sono relacionadas à idade podem desempenhar um papel importante na medida em que idosos saudáveis apresentam um declínio nas FE.

**Métodos:**

Uma estratégia de revisão sistemática foi empregada para identificar a literatura disponível sobre mudanças no sono relacionadas ao envelhecimento e FE.

**Resultados:**

Dos 465 estudos identificados, 26 foram incluídos. Os resultados sugerem que múltiplos parâmetros do sono diferem na forma como beneficiam ou comprometem as FE. Parâmetros como maiores despertares após o início do sono e baixa eficiência do sono, além da fragmentação circadiana do sono, mostraram resultados mais consistentes e potencialmente se correlacionaram com a piora nas medidas de FE. Contudo, outros resultados parecem inconclusivos.

**Conclusão:**

Esses achados foram discutidos baseadamente no modelo de vulnerabilidade pré-frontal, no qual o sono tem sido apontado como um fator benéfico para o funcionamento do córtex pré-frontal e, consequentemente, para as FE que têm como substrato principal essa área cerebral e regiões relacionadas.

## INTRODUCTION

The process of normal aging affects the sleep-wake regulatory system in ultradian, circadian and homeostatic levels. Changes in ultradian rhythm of sleep and sleep parameters include decreases in slow wave sleep (SWS, stage N3), which appear to begin as early as midlife, as well as decrease in total sleep time (TST) and increased wake after sleep onset (WASO), the latter resulting in poor sleep efficiency.^1^ ^-^ ^3^ The percentage of SWS linearly decreases at a rate of approximately 2 per cent per decade up to 60 years and then stabilizes through the mid-90s.^4^ ^,^ ^5^

Older adults spend an increasing percentage of sleep in stages N1 and N2, resulting in less restorative sleep.^6^ Crowley, Trinder, Kim, Carrington & Colrain found that sleep spindle number, density and duration, as well as K-complex number and density, are lower in younger than in older adults.^7^ A slight decrease in percentage of rapid eye movement (REM) sleep may occur, decaying by less than 1% per decade or may remain relatively unchanged.^8^

Regarding circadian organization of sleep in elderly, there is commonly a phase advance in the circadian sleep cycle: a propensity toward an earlier sleep onset, together with an earlier morning wake pattern.^9^ In other words, older people become sleepy in the evening and tend to sleep quite early, hence waking up earlier in the morning.^10^ This advance in sleep phase is complicated by social pressure to stay up later in the evening, despite their altered internal rhythm. 

There are also changes in homeostatic organization of sleep: it has been hypothesized that in the elderly, homeostatic sleep pressure undergoes a decrease and may be a cause of reduced TST, SWS and sleep efficiency.^11^ ^,^ ^12^ This theory is evidenced by an increased amount of nocturnal awakenings and diminished daytime sleepiness in elderly compared to young adults.^13^ Despite this reduced daytime sleepiness, the coexistence of a high frequency of daytime napping seems paradoxical. Up to 60% of older adults takes a nap,^14^ most likely resulting from lifestyle factors, such as opportunities to nap due to schedule flexibility as a result of retirement, maladaptive habits or even medication side effects. Although considered non-pathological, the ultradian, circadian and homeostatic age-related changes mentioned above may lead to complaints in the elderly and, when combined with lifestyle factors, lead to poor sleep quality or sleep deprivation. 

Healthy aging also involves changes in cognitive functioning. Thereby a certain amount of cognitive decline is a normal part of aging, varying considerably across individuals and cognitive domains, with some cognitive functions appearing more susceptible than others to the effects of aging.^15^ Declines in cognition tend to be most marked in executive functions (EF).^16^ ^,^ ^17^ EF is an umbrella term denoting a branch of top-down processes involved in the coordination and control of goal-directed behavior.^18^ ^-^ ^20^ Diamond^21^ describes three core executive functions: (1) inhibitory control (the ability to restrain one's habitual responses to override strong internal predispositions or external draw); (2) working memory (the ability to hold information in mind to support the completion of tasks); and (3) cognitive flexibility (the ability to ponder multiple sources and forms of information at one time and adaptively switch between them when engaging in a task). From these core functions, other EF functions are derived, such as planning, reasoning, problem-solving. However, other EF are also considered in other models, such as the ability to sustain attention, resistance to interference, utilization of feedback, multitasking, and verbal fluency.^19^ Adequate executive functioning is necessary for selecting and monitoring actions that facilitate the fulfillment of chosen goals and supports older adults to perform complex activities in everyday life.^22^

Changes in executive functioning related to healthy aging can be explained at least partially by structural and functional changes in the central nervous system. Although the loss of neurons in the elderly is moderate,^23^ studies have shown that loss of neuronal and dendritic architecture, rather than loss of neurons, underlies neocortical volume loss with increasing healthy age.^24^ Changes in frontal-striatal circuits are the most likely significant cause of reduced EF in non-mild cognitive impairment (MCI) and nondemented older adults.^25^ Frontal-striatal systems are preferentially vulnerable to white matter change, atrophy, and certain forms of neurotransmitter depletion.^25^ ^-^ ^27^ Frontal-striatal and frontoparietal circuitry play an important role in executive functioning.^28^ ^,^ ^29^

Given that both sleep components and EF undergo alterations in healthy aging, associations between these factors have been considered, as well as the impact of sleep changes (i.e. ultradian, circadian and homeostatic) in prefrontal circuits that underlie EF. Horne & Harrison first hypothesized that the waking function of the prefrontal cortex (PFC) and the frontal predominance of EEG delta activity in sleep may be linked.^30^ ^-^ ^32^ According to this model and more recent findings, sleep loss selectively affects the efficiency of PFC circuitry, producing changes in brain metabolism and affecting executive functioning.^33^ ^-^ ^35^ Since then, some studies have argued that cognitive functioning related to the PFC is particularly vulnerable to sleep loss or sleep deprivation.^36^ ^,^ ^37^

Assuming the prefrontal circuit vulnerability to sleep loss hypothesis and the parallel decreases in sleep and EF in older adults, the present article provides a systematic review of evidence of interaction between these two functions in healthy aging, that is, a growing body of literature suggesting that changes in sleep may play an important role in the extent to which healthy older adults exhibit decreases in executive functioning.

## METHOD

**Figure 1:**
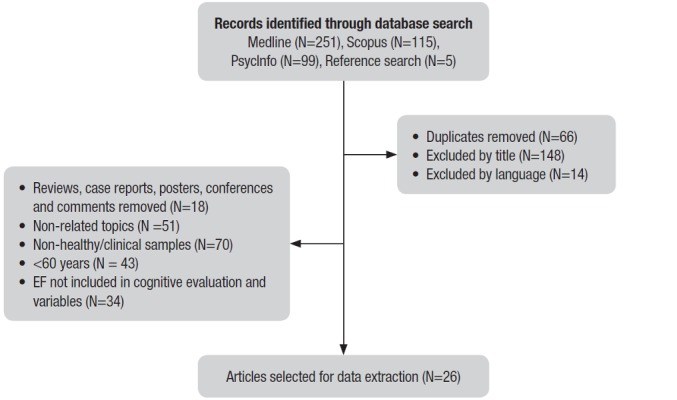
Flow-chart describing process of study selection.

Search strategy. A systematic strategy was employed to identify the available literature on age-related sleep changes and EF. Search terms included: (executive funct*) AND (sleep) AND (aging OR older adults OR elderly). The following databases were searched using these terms: Medline, PsycInfo and Scopus. Search terms were systematically applied across these three databases. The numbers of relevant hits from these databases are summarized in Figure 1. The retrieved articles were referenced in EndNote (Version 17.0) and duplicates removed. This search strategy was augmented with hand searches of reference lists of included studies. Searches were conducted on 10^th^ May 2016. 

Study selection. Inclusion and exclusion criteria were outlined prior to the search. Inclusion criteria included: 1) articles published in the last 25 years (1990-2016); 2) sample comprising healthy older adults (60 years or older, without dementia, mild cognitive impairment, sleep disorders or loss of functionality); 3) empirical research; and 4) focus on sleep variables (e.g. ultradian, circadian and homeostatic), healthy aging and cognitive functioning, addressing EF or at least one EF domain evaluated. The exclusion criteria established were: 1) case studies, letters to the editor, and conference abstracts; 2) non-human experimental model studies; 3) non-healthy/clinical samples of older adults, such as diagnosed with sleep disorders, dementia and cognitive impairment; 4) EF not included in cognitive evaluation and variables; 5) non-English language; and 6) review articles.

The 465 articles initially retrieved from the preceding detailed searches were screened by their titles and abstracts, and articles not meeting the inclusion and/or exclusion criteria described earlier were removed. This gave a total of 26 articles eligible for review. 

## RESULTS

We included 26 articles in the review. A summary and analysis of these articles are given in Table 1. In general, the studies varied widely in their research design, participants and sleep and EF variables. A large portion of the studies were conducted by researchers in the United States (n=16). This was followed by the United Kingdom (n=2), France (n=2), the Netherlands (n=2), Canada (n=1), Ireland (n=1), Japan (n=1), and Switzerland (n=1). In relation to study populations, all the articles in this review were based on non-clinical elderly samples, most of them including home/community-dwelling subjects. The main sleep variables included in articles were WASO, sleep latency, sleep efficiency, TST, sleep quality and other less frequent variables such as sleep deprivation and sleep stages. Sleep circadian and homeostatic influences were also included in some studies. 

**Table 1:** Overview of studies assessing the impact and the relation of sleep and executive functioning.

Reference*	Sample	Instruments appplied	Evaluated variables of EF and sleep	Main results	Main limitations
Anderson & Horne^60^	N = 24 (10 men and 14 women) Mean age 67 years	EF: WCST, Tower of London, Verbal fluency task, Cattell Test of Fluid Intelligence Sleep: Sleep EEG	EF: Planning, flexibility, verbal fluency, fluid intelligence Sleep: Non REM period	The study found significant associations between 0.5-1.0 Hz power from the left frontal EEG channel, in the first non-REM period, and performance at tasks more specific to the left PFC (e.g., nonverbal planning and verbal fluency).	Age range and number of participants relatively small.
Blackwell et al.^38^	N = 2932 (women) Mean age 83.5 years	EF: TMT (part B) Sleep: Actigraphy	EF: Cognitive flexibility Sleep: WASO, sleep latency, sleep efficiency, total nap time, TST	Compared with women with sleep efficiency >70%, those with <70% had a higher risk of cognitive impairment. Higher sleep latency was associated with higher risk of cognitive impairment, as was higher WASO. There was no significant relationship for TST.	Findings are for older women and may not be generalizable to other populations such as men.
Schmutte et al.^50^	N = 375 (134 men and 241 women) Mean age 79.6 years	EF: Similarities (WAIS III), Digit span (WAIS III), Block design (WAIS III), DSST (WAIS III) Sleep: 54-item sleep questionnaire	EF: Abstract reasoning, working memory, visual-spatial reasoning, processing speed Sleep: Self-reported sleep duration, self-onset latency	Participants who reported longer sleep onset latencies performed significantly worse on measures of verbal knowledge, long-term memory and fund of information, and visuospatial reasoning. Participants who reported longer sleep durations did significantly worse on a measure of verbal short-term (working) memory.	The sleep measure created for the study has no documented psychometric properties. Absence of any objective corroborating data such as PSG.
Yaffe et al.^39^	N = 2474 (women) Mean age 68.9 years	EF: TMT (part B) Sleep: Actigraphy	EF: Cognitive flexibility Sleep: WASO, sleep latency, sleep efficiency, total nap time, TST	Women who declined on Trails B experienced worse sleep efficiency, sleep latency, and wake after sleep onset. Women who declined on Trails B napped more. There was not association with total sleep time.	Actigraphy was performed only at follow-up. Cognitive battery was somewhat limited and only included measures of global cognition and EF.
Gamaldo et al.^53^	N = 174 (51 men and 123 women) Mean age 72.7 years	EF: Backward digit span task, Alpha Span task Sleep: Self-reported item asking about trouble falling asleep	EF: Working memory Sleep: Self-reported trouble falling asleep	Self-reported sleep trouble significantly predicted performance on the digit span and alpha span task.	Limited assessment of performance. Self-reported item asking about trouble falling asleep does not account for other possible sleep problems.
Oosterman et al.^56^	N = 144 (90 men and 54 women) Mean age 69.5 years	EF: Digit span backward (WAIS III), Stroop test, TMT (part B) Sleep: Actigraphy	EF: Working memory, inhibition, cognitive flexibility Sleep: Sleep fragmentation	The fragmentation of the sleep-wake rhythm predicted all cognitive functions examined. Partial correlations showed that the association of rhythm fragmentation with cognitive decline is partly independent from main effects of age.	Majority of the subjects suffered from at least one cardiovascular risk factor. No objective screening was performed to examine the possible presence of SDB.
Nebes et al.^48^	N = 157 (gender not reported) Mean age 72 years	EF: Perceptual comparison task, N-back, Letter-Number Sequencing (WAIS III), Stroop test, Hayling test, TMT (part B), Test of Nonverbal Intelligence III Sleep: PSQI	EF: Information processing speed, working memory, inhibitory function, attention shifting, abstract reasoning Sleep: Subjective sleep quality, sleep latency, sleep duration, sleep efficiency	Poor sleepers performed significantly worse than good sleepers on measures of general neuropsychological status, abstract reasoning, attention shifting and working memory. It was found that sleep latency and efficiency were correlated with cognitive performance, whereas total sleep duration was not.	Sleep was assessed with a general retrospective self-report measure. There was also no measure of SDB.
Gamaldo et al.^52^	N = 50 (11 men and 39 women) Mean age 65.4 years	EF: Stroop test, Clock Drawing Test, Letter Series Test, Letter Fluency. Sleep: PSQI	EF: Inhibition, inductive reasoning, verbal fluency, global executive functioning Sleep: Sleep quality and sleep habits.	A within-person daily change in sleep duration was associated with worse global cognitive performance. The greater an individual deviated away from his/her average sleep duration on a particular day, the more likely his/her performance would decline.	Small sample of only a homogenous group of African American elders. The study relied on subjective rather than objective assessments of sleep.
Blackwell et al.^40^	N = 3132 (men) Mean age 76.4 years	EF: TMT (part B) Sleep: Actigraphy, Sleep diary, PSQI, EES	EF: Mental flexibility Sleep: TST, sleep efficiency, WASO, number of long wake episodes, subjective sleep quality, subjective daytime sleepiness	There were modest cross-sectional associations of wake after sleep onset and self-reported long sleep with cognition among older community dwelling men. Excessive daytime sleepiness and self-reported poor sleep were not related to cognition.	The findings may not be generalizable to populations groups other than community-dwelling older men. Causality cannot be established due to the cross-sectional study design.
Saint Martin et al.^45^	N = 272 (79 men and 193 women) Mean age 74.8 years	EF: TMT (A and B), Code test (WAIS-III), Similarity test (WAIS-III), Stroop test, Alphabetic Fluency and Category Fluency Tasks, Benton Visual Retention Test (form C) Sleep: PSQI, EES	EF: Attention, shift capacity, rule maintenance capacity, abstractive reasoning, inhibition, verbal fluency, visuospatial working memory Sleep: Subjective sleep quality, excessive daytime sleepiness, sleep duration, sleep latency	Subjective sleep quality and its duration in healthy elderly showed no significant influence on cognitive performance (subjective and objective), except the attention level.	Sleep duration and quality were self-reported and it had no information on sleep structure and sleep fragmentation.
Sagaspe et al.^59^	N = 11 (men) Mean age 68 years	EF: Go/noGo task, Simple Reaction Time Task Sleep: Actigraphy, visual analogue scale sleepiness	EF: Inhibitory motor control, sustained attention Sleep: Sleepiness, sleep deprivation	In the sleep deprivation condition, inhibitory motor control was impaired by extended wakefulness equally in both age groups (young and male). Sustained attention on the executive task decreased under sleep deprivation in both groups, and even more in young participants.	It was not presented.
Sutter et al.^51^	N = 107 (46 men and 61 women) Mean age 72 years	EF: Regensburg Word Fluency Test, DSST, Subtest 3 of the German Achievement Measure System, Tests of Attentional Performance, TMT (A and B) Sleep: PSQI	EF: Verbal fluency, processing speed, reasoning, inhibition, set-shifting Sleep: Self-reported sleep quality.	Poorer sleep quality was associated with lower performance in reasoning, semantic fluency, and shifting in those with high versus low levels of subclinical depression. Poor sleep quality might affect higher order cognitive processes, particularly in those reporting higher levels of subclinical depression.	Measured each cognitive domain with just one or two single cognitive tests. It did not assess the detailed usage of non-psychoactive medications. The study relied on subjective e assessments of sleep.
Zimmerman et al.^54^	N = 549 (208 men and 341 women) Mean age 79.7 years	EF: TMT (part B), Category and Letter Fluency test. Sleep: Medical Outcomes Study Sleep Scale	EF: Mental flexibility, set-shifting, concept formation, verbal fluency Sleep: Sleep initiation, sleep maintenance	Older adults with lower education appear selectively vulnerable to the negative effects of sleep onset/maintenance difficulties on tests of verbal fluency.	Determination of sleep onset/maintenance difficulties was based on a self- report questionnaire.
McCrae et al.^44^	N = 72 (gender not reported) Mean age 70.1 years	EF: Letter Series total Sleep: Sleep diary	EF: Inductive reasoning Sleep: TST, WASO	TST did not predict executive functioning or processing speed. Total wake time did not predict executive functioning but significantly predicted processing speed.	Study's relatively small sample size.
Lim et al.^57^	N = 700 (172 men and 528 women) Mean age 82.4 years	EF: Digit span test, Digit ordering test Sleep: Actgraphy	EF: Working memory Sleep: Fragmentation of rest and activity	Greater fragmentation of rest and activity were associated with lower levels of cognitive performance, with preferential involvement of perceptual speed, semantic memory, working memory, and visuospatial abilities.	Primarily women aged 80 and over.
Miyata et al.^43^	N = 78 (16 men and 62 women) Mean age 72.2	EF: Number (n)-back test Sleep: Actigraphy, PSQI, EES	EF: Working memory Sleep: TST, WASO, sleep efficiency, sleep latency, daytime sleepiness	Short sleep duration decreased short-term memory capacity. Participants with sleep efficiency <85% showed a significant decrease on short-term memory and working memory test accuracy compared with those with sleep efficiency >85%.	The study included a high percentage of female participants.
Wilckens et al.^41^	N = 45 (13 men and 32 women) Mean age 62.8 years	EF: Sternberg working-memory task, N-back task, Stroop task, Flanker task, National Adult Reading Test (NART), Categorical and Lexical Fluency tasks Sleep: Sleep detection device	EF: Working memory, inhibition, verbal fluency and proficiency Sleep: WASO, TST	In the older group, higher sleep continuity was associated with better inhibitory control, memory recall, and verbal fluency. TST was not associated with cognitive performance in any domains for the older group.	Participants were not excluded based on any sleep measures or sleep disorders. The study used an accelerometer-based sleep detection device, whereas PSG is considered the "gold standard" for sleep measurement.
Wilckens et al. ^42^	N = 53 (gender not reported) Mean age 62.6	EF: TMT (A and B), DSST (WAIS-III), Stroop Task, N-Back, task-switching Sleep: A sleep detection device	EF: Attention, cognitive flexibility, working memory, inhibition Sleep: WASO, TST	Better global switching performance was associated with longer and more continuous sleep. Young and older adults may benefit similarly from lower wake time after sleep onset and longer total sleep time in overall performance.	The study used an accelerometer-based sleep detection device, whereas PSG is considered the "gold standard" for sleep measurement.
McHugh et al.^55^	N = 505 (gender not reported) Mean age 73.4 years	EF: Digit span backward, CAMCOG similarities, TMT (A and B) Sleep: PSQI	EF: Divided attention, working attention, psychomotor speed Sleep: Time to bed, time do rise	Early and late sleepers were significantly slower on attention, learning and praxis tasks than those whose bedtime did not differ significantly to the robust norm. Wake-times were not associated with cognitive functioning in this cohort.	Self-report measures of sleep. There were no guidelines to categorise morningness-eveningness behavior of a less extreme type among otherwise healthy older adults.
Groeger et al.^62^	N= 31 (6 men and 25 women) Mean age 70.8 years	EF: DSST, Sustained attention to Response Task, Choice Reaction Time Test, Lexical Decision Time, Serial Reaction Task, Continuous Tracking Task, Pursuit Tracking Task,Verbal n-Back and Spatial n-Back, Goal Neglect Task, Paced Visual Serial Addition Task, Verbal Fluency Task Sleep: PSG, PSQI	EF: Sustained attention, divided attention, processing speed, decision, sequence & motor control, working memory, verbal fluency. Sleep: Slow wave sleep disruption	Slow wave sleep disruption resulted in less positive affect, slower or impaired information processing and sustained attention, less precise motor control, and erroneous implementation, rather than inhibition, of well-practiced actions. At baseline, younger participants performed better than older participants across many cognitive domains, with largest effects on executive function, response time, sustained attention, and motor control.	It was not presented.
Walsh et al.^58^	N = 1287 (women) Mean age 82.8 years	EF: Digits Span Backwards (WAIS III); TMT (part B), Categorial and letter fluency Sleep: Actigraphy, PSQI, EES	EF: Working memory, task-switching, attention, verbal production Sleep: Amplitude, mesor, rhythm robustness, acrophase, subjective sleep duration; TST, subjective sleepiness	Weaker circadian activity rhythm patterns were associated with worse cognitive function, especially executive function, in older women without dementia.	Limited to a single sex and primarily Caucasian population. The study did not have detailed cognitive function tests at baseline to control for initial differences in executive function.
Lambiase et al.^49^	N = 121 (women) Mean age 73.3 years	EF: DSST, TMT (A and B), Verbal fluency task Sleep: Actigraphy, Sleep diary	EF: Attention, psychomotor speed, set shifting, mental flexibility, verbal fluency Sleep: TST, sleep efficiency, time to go to bed, time to wake up, sleep latency, number and minutes of awakenings	Sleep efficiency was associated with more correct responses on the DSTT. Sleep was not associated with verbal fluency. Lower sleep efficiency was associated with poorer performance on both the DSST and the TMT B among women with low levels of physical activity but not among women with high levels of physical activity.	The sample was primarily white, well-educated, older women with good overall cognition. The week may not be reflective of habitual sleep or physical activity patterns of the participants.
Lafortune et al.^61^	N = 58 (33 men and 25 women) Mean age 63 years	EF: The Bells Test, Conners' Continuous Performance Test II, Verbal fluency task, N-Back task Sleep: PSG	EF: Selective visual attention, inhibition, verbal fluency, working memory Sleep: Spindles, slow wave, sleep latency, REM latency, sleep duration, sleep efficiency, sleep stages (duration)	Spindle density in healthy middle-aged and older participants predicted verbal learning, visual attention and verbal fluency performance. Slow wave density and slow wave slope predicted verbal fluency performance only.	Significant correlations had mild to moderate effect sizes. The first-night effect was not controlled in this study.
Luik et al. ^47^	N = 1723 (810 men and 913 women) Mean age 62 years	EF: Letter digit substitution task, Stroop color word test Sleep: Actigraphy, Sleep diary	EF: Sustained attention, psychomotor speed, mental flexibility, inhibition Sleep: Duration actigraphy, interdaily stability, intradaily variability, sleep-onset latency, total sleep time, perceived sleep quality	Persons with less stable 24-h rhythms performed worse on the letter digit substitution task and the stroop interference trial after full adjustment. Similarly, persons with more fragmented rhythms performed worse on the letter digit substitution task and the stroop. Longer observed sleep onset latencies were related to worse performance on the word listening test delayed recall and the categorical word fluency test.	Actigraphy allows to estimate sleep parameters, but it lacks the precision of PSG. The study did not formally assess chronotype. Self-rated but no objective information about sleep-disordered breathing.
Seelye et al.^46^	N = 63 (11 men and 52 women) Mean age 87 years	EF: Letter-Number Sequencing (WMS III), Digit span (forward and backward) (WAIS R), DSST (WAIS R), TMT (Part A and B), Letter fluency, Stroop test Sleep: Sensor-based sleep assessment	EF: Working memory, attention, cognitive flexibility, verbal fluency, inhibition Sleep: Total movement in bed at night, restlessness, times up at night, TST	Mildly disturbed sleep the week prior and month prior to cognitive testing was associated with reduced working memory on cognitive evaluation. One night of mild sleep disturbance was not associated with decreased cognitive performance the next day. Sleep duration was unrelated to cognition.	The number of individuals with diagnosed sleep disorders was unknown.
Song et al.^63^	N = 2601 (men) Mean age 76 years	EF: TMT (Part B) Sleep: PSG	EF: Cognitive flexibility Sleep: Sleep stages	Increased time in Stage N1 sleep and less time in Stage REM sleep are associated with worsening cognitive performance in older men over time.	Participants had relatively high levels of cognitive function at baseline (sleep visit) and follow-up. First-night effect caused possibly by single overnight PSG.

*Studies are presented in order of year of publication. EF: executive functions; TMT: Traik Making Test; WCST: Wisconsin Card-Sorting Task; WAIS: Wechsler Adult Intelligence Scale; WASO: wake after sleep onset; TST: total sleep time; SDB: sleep disordered breathing; PSQI: Pittsburgh Sleep Quality Index; DSST: Digit Symbol Substitution Test; PSG: polysomnography; ESS: Epworth Sleepiness Scale

A total of 10 studies (n=9094) evaluated sleep using objective measures only. Seven studies (n=6383) were based on objective and subjective measures. A total of 9 studies (n=2261) evaluated sleep using subjective measures only. Actigraphy was the most used objective measure by studies (n=10), followed by polysomnography (PSG) (n=4) and other sleep detection devices (n=3). The most common subjective measure was the Pittsburgh Sleep Quality Index (PSQI). Others included the Epworth sleepiness scale (ESS) (n=4), sleep diary (n=4) and other sleep questionnaires (n=3). A large variety of tests and instruments were used to assess EF. The Trail Making Test (TMT) was used in more than half of the studies (n=14). Other more frequent measures were Verbal Fluency Tasks (n=11), the Stroop test (n=7), Digit Span Test (n=7), *n*-back Task (n=6), and Digit Symbol Substitution Test (DSST) (n=6). Other less frequent tests used are given in Table 1.

Wake after sleep onset (WASO). WASO means the total amount of time awake after falling sleep and it is considered a better reflection of sleep fragmentation. There is evidence that WASO could be related to executive functioning and global cognition. Using actigraphy, Blackwell et al.^38^ found that higher WASO was associated with higher risk of cognitive impairment, using the TMT as a measure of executive functioning and the Mini-Mental State Examination (MMSE) for global cognition. In a longitudinal community-based study by Yaffe et al.^39^ elderly women who showed decline on the TMT (part B) experienced worse WASO. Using the same measures, another study by Blackwell et al.^40^ found a modest association. Recently, in two studies Wilckens et al.^41^ ^,^ ^42^ found that in older adults, higher sleep continuity (i.e. lower WASO) was associated with better inhibitory control and that individuals with less WASO were more likely to engage preparatory strategies to reduce switch costs and boost task-switching performance. These findings suggest that a higher WASO negatively impacts executive functioning. Inversely, older adults may benefit from lower WASO in performing complex tasks.

In another study, however, although participants with WASO longer than 30 min tended to have lower accuracy on the *n*-back test (attention and working memory measure) than those with WASO <5 min or 5-30 min, these differences were not significant.^43^ McCrae et al.^44^ found that WASO did not predict executive functioning (measured by a reasoning test) but significantly predicted processing speed. This seems to indicate that in some cases, sleep fragmentation primarily affects basic processes, such as processing speed and alertness, rather than more complex ones.

Total sleep time (TST). In some studies, TST was examined as a continuous variable while in other studies it was dichotomized into short and long sleep duration. The results of its impact on EF and other cognitive domains are controversial. Many studies found no association between objectively measured total sleep duration and executive functioning.^38^ ^-^ ^40^ ^,^ ^42^ ^,^ ^46^ ^,^ ^47^ This lack of relationship was also detected in subjectively measured total sleep duration.^44^ ^,^ ^45^ ^,^ ^48^ Miyata et al.^43^ found that TST was correlated with the 0-back test from the *n*-back test, a simple measure of attention and short-term memory, but not for the 1-back test, which reflects working memory capacity.

Nevertheless, in the study by Wilckens et al.^41^ a better performance on task-switching, a model paradigm of executive functioning that involves cognitive flexibility and the ability to shift attention between one task and another, was associated with longer TST. There have been cases in which subjective and objective measures diverged in associations: Lambiase et al.^49^ reported that total sleep duration measured using a sleep diary, a subjective instrument, was associated with cognitive flexibility; on the other hand, in the same study, actigraphy measures did not show this association. Taken together, these studies of sleep duration and executive functioning in older adults have produced mixed results, although most of the evidence mentioned above indicates no relationship between them and may suggest that it is interruption of sleep, such as higher WASO, rather than quantity, that most affects EF. 

Sleep latency. In relation to sleep latency, defined as the length of time that it takes to accomplish the transition from wakefulness to sleep, the reviewed articles reported mixed results. When examining objective reports, Blackwell et al.,^38^ Yaffe et al.^39^ and Blackwell et al.^40^ showed that higher sleep latency was associated with worse executive functioning based on performance on the TMT (part B). Schmutte et al.^50^ reported that longer self-reported sleep latency was significantly and inversely related to verbal-based cognitive measures, abstract reasoning, and longer latencies associated with poorer cognitive functioning. Nebes et al.^48^ also showed that a longer self-reported time to fall asleep was associated with poorer abstract reasoning. Others reported lack of association between sleep latency and EF through subjective^45^ and objective^43^ measures. It may be that longer sleep latency disturbs sleep quality and quantity and thus exacerbates the negative impacts on EF. 

Sleep efficiency. Sleep efficiency, defined as the percentage of total time in bed actually spent in sleep, seems to impact EF. Blackwell et al.^38^ found that compared with women that had a sleep efficiency >70%, those with <70% had a higher risk of cognitive impairment measured by the TMT (part B) for executive functioning and by the MMSE for global cognition. In addition, older women who showed decline on the TMT (part B) had worse sleep efficiency.^39^ Participants with sleep efficiency <85% showed a significant decrease in 0- and 1-back test accuracy compared to individuals with sleep efficiency ≥85%, i.e. as objective sleep efficiency decreased attention and working memory worsened.^43^ Lambiase et al.^49^ also reported that lower actigraphy-assessed sleep efficiency was associated with poorer performance on executive function tasks of attention, set-shifting and cognitive flexibility. On subjective measures, sleep efficiency was correlated with measures of abstract reasoning and working memory.^48^ Nevertheless, using the same measures as Blackwell et al.^38^ and Yaffe et al.,^39^ Blackwell et al.^40^ failed to find these associations. According to the authors, this less consistent finding observed for sleep efficiency may relate to greater measurement error of this variable. Overall, the studies indicated that older adults with lower percentage sleep efficiency exhibited worse executive functioning. 

Sleep quality. Sleep quality does not refer only to the number of hours in bed. It is the quality of these hours and how other factors affect sleep that are important, such as fragmentation, sleep deprivation, perception of restorative sleep, daytime sleepiness, having trouble falling sleep and waking up in the morning or staying alert during the daytime. Most of the studies used the PSQI as a general measure of sleep quality. The results in the literature regarding the effect of this parameter on EF tend to be mixed. In older women, good (PSQI < 6) and poor (PSQI ≥ 6) sleepers differed significantly on tests of working memory, attention, set shifting, and abstract problem solving,^48^ suggesting an important role of sleep quality in executive functioning. Sutter et al.^51^ sought to clarify the relationship between sleep quality and cognitive performance in healthy older adults, and to evaluate the moderating role of subclinical depression in this relationship. The study found that self-reported sleep quality in healthy older adults seemed to be selectively related to higher order executive functions in those participants with high versus low levels of subclinical depression.

In other lines of evidence, these effects were not found. Gamaldo et al.^52^ used inhibition, inductive reasoning, verbal fluency and global executive functioning together with other cognitive domains, to create a global composite score and found that sleep quality measured by PSQI was not a significant predictor of performance across cognitive domains. Although Blackwell et al.^40^ reported that almost half of the men (44%) had self-reported poor sleep quality, defined as PSQI > 5, no significant association between this subjective measure and executive functioning was confirmed. Also, excessive daytime sleepiness did not correlate with cognitive outcomes. Martin et al.^45^ observed that good and poor sleepers did not differ on any of the cognitive function measures, including those evaluating EF domains, except on the TMT (part A), where this latter result may reflect attention impairment. Luik et al.^47^ also found no relationship between lower reported sleep quality, as measured by a sleep diary and global cognitive functioning or performance on specific cognitive tasks, such as inhibition. Taken together, these contrary findings do not seem to confirm the relationship between sleep quality and EF. 

Some articles reviewed also used other variables related to sleep quality. Gamaldo et al.^53^ examined the relationship between elders' cognitive performance and self-reported trouble falling asleep and found that this complaint significantly predicted performance on the digit span and alpha span task (measures of working memory and attention). This demonstrates that a self-report of sleep difficulty may be a predictor of cognitive performance. Zimmermann et al.^54^ reported that sleep difficulties (i.e. sleep initiation and sleep maintenance) appear to selectively impact the verbal fluency process in older adults with lower education measured by category fluency and letter fluency tests, suggesting that those with higher education are better able to engage active cognitive compensation against sleep difficulties than individuals with lower education. 

Circadian, homeostatic and ultradian factors. Another study found an association between time to bed and cognitive functioning, independent of sleep duration. Older adults who went to bed much later showed a poor performance on the TMT (A and B) and on the drawing test (prefrontally-mediated tasks) compared to older adults who went to bed within the robust norm window.^55^ Thus, it appears that severe deviations in time to bed represent a marker of circadian misalignment and a possible marker of cognitive impairment. The study by Oosterman et al.^56^ reported that fragmentation of the sleep-wake rhythm predicted neuropsychological functioning such as mental speed, verbal memory and EF (cognitive flexibility, interference and working memory) in home-dwelling elderly people. Partial correlations showed that the association of rhythm fragmentation with cognitive decline is partly independent of the main effects of age. Therefore, part of age-related cognitive decline could independently be associated with sleep and its circadian organization. Another study found that greater fragmentation of rest and activity measured by actigraphy was associated with lower levels of cognitive performance, including working memory.^57^

A prospective observational study in a large cohort of older adult women without dementia showed that weaker circadian activity rhythm patterns (i.e. amplitude, mesor, rhythm robustness, and acrophase) were associated with worse cognitive function, especially executive functioning measured by the TMT (part B).^58^ Disrupted circadian activity rhythms could be an early indicator of future executive function decline. In another study, actigraphy recordings were used to quantify 24-h rhythms by calculating the stability and fragmentation of the rhythm over a period of days. Both aspects of the 24-h activity rhythm and sleep parameters were related to global cognitive functioning, but specifically, disturbances in the 24-h activity rhythm were mostly related to tasks that draw on perceptual speed and executive functioning (mental flexibility and inhibition).^47^ Perhaps his association could reflect a direct effect of disturbed rhythms on perceptual speed and executive functioning. 

Sagaspe et al.^59^ evaluated inhibitory motor control and sustained attention under controlled high or low sleep pressure conditions in young and older males. Under the sleep deprivation condition, inhibitory motor control was equally impaired by extended wakefulness in both age groups. Although sustained attention also decreased under sleep deprivation conditions in both groups, this effect was more pronounced for young participants. This might indicate that aging is a protective factor against the effects of extended wakefulness on simple tasks (i.e. sustained attention) due to an attenuation of sleep pressure with duration of time awake. In other words, homeostatic sleep pressure would be lower in the older people, allowing them to be less vulnerable to sustained attentional failure after a night of sleep deprivation. 

Lastly, some of the studies reviewed focused on sleep stages and sleep EGG measures. Anderson & Horne^60^ examined associations between neuropsychological performance, such as planning, flexibility, verbal fluency, and fluid intelligence, and sleep EEG characteristics within the prefrontal cortex in 24 healthy 61-75-year-olds. They found significant associations between 0.5-1.0 Hz power from the left frontal EEG channel, in the first non-REM period, and performance on tasks more specific to the left PFC (e.g., nonverbal planning and verbal fluency). These results pointed to a sleep EEG correlate of neuropsychological performance centering on the PFC. Lafortune et al.^61^ found that spindle density in healthy middle-aged and older participants predicted verbal learning, visual attention and verbal fluency performance. Slow wave density and slow wave slope predicted verbal fluency performance only. These results suggest that spindle density is a marker of cognitive functioning in older adults and may reflect neuroanatomic integrity.

Groeger et al.^62^ assessed the effects of reducing SWS on daytime functioning and whether these effects differ across groups of healthy young, middle-aged, and older individuals. Evaluating domains such as sustained and divided attention processing speed, decision, sequence and motor control, working memory, and verbal fluency, the study showed that SWS disruption resulted in slower or impaired information processing and sustained attention, less precise motor control, and erroneous implementation, rather than inhibition, of well-practiced actions. Younger participants performed better at baseline than older participants across many cognitive domains, with largest effects on executive function, response time, sustained attention, and motor control. SWS can possibly be considered a potential mediator of age-related decline in performances. Song et al.^63^ investigated the relationship between sleep stage distributions and subsequent decline in cognitive function in older men over time, using the TMT (part B) as a measure of executive functioning and the Modified Mini-Mental State Examination (3MS) for global measurement of cognitive function. Increased time in stage N1sleep (considered a "light" sleep and marker of poorer sleep quality) and less time in stage REM sleep were associated with worsening general cognitive and executive performance in older men, suggesting a beneficial role of REM sleep in cognition. 

## DISCUSSION

The aim of the present review was to systematically understand the evidence of interaction between sleep and executive functions in healthy aging, considering a growing body of literature suggesting that changes in sleep parameters and in its ultradian, circadian and homeostatic organization may play an important role in the executive functioning. In the elderly population, however, the evidence for this association reviewed tends to be mixed and involves various inconsistencies. Multiple sleep parameters appear to differ, with some benefiting and others impairing executive functioning. 

Most of the studies found evidence for the association between WASO and EF and between sleep efficiency and EF. Both sleep parameters change with normal aging. WASO is a measure of time spent awake during the night. WASO time is a better reflection of sleep fragmentation and lower values indicate better sleep continuity during the night. Thus, elevated WASO directly affects sleep efficiency (proportion of total time in bed spent asleep). It is possible that higher WASO prevents older adults from progressing normally through sleep stages and attenuates the length of time spent in SWS.^41^ ^,^ ^42^ SWS and slow wave activity (SWA) have been identified as a beneficial factor for prefrontal cortex functioning and hence for executive functioning, which relies mostly on this brain area and its connections.^64^ ^,^ ^65^ Supporting lines of evidences include: (1) slow waves show frontal predominance both under baseline conditions and in response to sleep loss;^66^ ^,^ ^67^ (2) delta activity that is high during SWS is associated with cognitive performance;^68^ (3) selective SWS disruption is associated with poor cognitive performance, and even impaired executive functioning;^62^ (4) age-related medial prefrontal cortex gray matter atrophy has recently been shown to be associated with reduced SWA in older adults. Lower SWS was associated with reduced functional connectivity within PFC-hippocampal networks, suggesting that neural synchrony during sleep strengthens connections between the PFC and functionally related brain regions;^69^ (5) greater relative slow wave activity was associated with higher dorsolateral prefrontal metabolism.^65^ In addition, there is also migration of cortical activity patterns from the posterior (decreased at occipital and temporal channels) to anterior (increased at central and frontal channels) area after sleep deprivation.^70^

Thus, this growing body of evidence supports a potential role of certain aspects of sleep cerebral activity in benefiting prefrontal areas and its connections. This highlights the PFC vulnerability model which holds that sleep deprivation and poor sleep affect PFC circuitry, producing a mild, sub-clinical level of impairment.^32^ ^,^ ^33^ ^,^ ^71^ ^,^ ^72^ The cognitive processes supported by PFC-associated networks (i.e. frontal-striatal and frontoparietal), especially EF, seem to be the most sensitive to individual differences in sleep.^34^ ^,^ ^72^ This relationship may be strengthened considering that in normal aging both sleep and executive functioning change, which may produce a synergistic effect on the performance of executive tasks. 

High levels of sleep fragmentation (recurrent awakenings and/or stage shifts) may result in complaints of non-restorative sleep even when an apparently normal total sleep time is present. This may be a factor explaining why most studies reviewed here found inconsistent or mixed associations between TST and EF: interruption of sleep, such as higher sleep fragmentation and lower sleep efficiency appears to affect EF more than sleep quantity. It seems that continuous and consolidated sleep, which allows adequate progression through sleep stages and NREM-REM cycling, is important for executive functioning. 

Contrary to the PFC vulnerability model, other lines of evidence suggest that impaired sleep continuity and deprivation may cause sleepiness, which in turn may result in reduced vigilance and attentional failures. This reduced vigilance may negatively impact executive functioning. Therefore sleep could affect EF mediated by basic process impairment, such as vigilance, alertness and attention processes.^73^ In this case, we can only cautiously infer that older adults with poor sleep quality and sleep deprivation may be less alert during the day, which directly worsens their performance on executive functioning tasks. On the other hand, another potential factor is that it can be assumed that impairment in the executive performance of elderly caused by sleep loss only becomes apparent when the PFC areas of the brain are activated under excessively high demand.^51^

Age-related changes in sleep parameters and ultradian sleep factors do not act solely on executive functioning. The review also showed that the changes in circadian organization of wake-sleep cycle (i.e. time to bed as marker of sleep phase, rhythm fragmentation, and weaker circadian activity) and the homeostatic factors also appear to play an important role. The aging process appears to generate vulnerability to the impact of sleep and circadian rhythm disturbances on executive performance.^47^ By affecting both cognition and the rest-activity rhythm, age-related changes in brain structures may account for an association between these variables. Regarding homeostatic pressure, some results have indicated that an increase in errors on an executive task under extended wakefulness can be attributed mainly to the effect of sleep pressure with duration of time awake.^59^

Taken together, all of the relationships discussed above have important implications for clinicians and other health professionals, where psychoeducational and cognitive-behavioral interventions focusing on sleep hygiene and sleep parameters could improve global sleep and consequently may benefit cognitive functioning.^74^ Sleep quality in the elderly population is often poor not because of specific pathologies, but due to lifestyle factors, such as daytime napping, alcohol consumption, medication side effects, that detract from adequate nocturnal sleep. In these cases, psychoeducational and cognitive-behavioral interventions can help by reducing the effects of insufficient sleep on waking performance. In addition, higher physical activity levels can protect against the negative effects of poor sleep on executive functioning, and also promote other well-documented benefits of a physically active lifestyle.^49^

While the majority of studies address many cognitive domains, here we focused on EF only. Consequently, we did not explore the relationship between sleep and domains such as episodic memory and visuospatial abilities. Few studies focus solely on the relationship between sleep and executive functioning. Concerning limitations of the literature reviewed, some studies did not exclude participants based on measures of sleep disorders, for example examining the possible presence of sleep disordered breathing, a condition that impairs cognition in older adults. In addition, many studies relied on subjective rather than objective assessments of sleep. For example, self-reported items about trouble falling asleep or time of sleep duration do not account for other possible sleep problems and are highly dependent on participant's perception and beliefs about sleep, which may lead to differential misclassification and selective drop-out. For example, polysomnography is considered the "gold standard" for sleep measurement and may shed light on brain activity during sleep and on its role in cognitive functioning. Covariables such as depression, anxiety, and medical illness should also be accounted for when both sleep and EF are investigated. Another important consideration is that most of the studies used relatively few EF tests and tasks. Further studies should employ a more comprehensive range of EF tasks and components to establish a better association between sleep and executive functioning. 

In conclusion, healthy aging is marked by changes in both sleep and executive functioning. Although evidence of the role of sleep and its ultradian, circadian and homeostatic organization in EF has grown, the association between them is partially inconclusive and studies using valid and reliable measures for sleep and EF variables are clearly needed. However, sleep parameters such as wake after sleep onset and sleep efficiency, in addition to circadian fragmentation of sleep, showed less mixed results and are potentially correlated with EF measures. This relationship has important implications for clinicians and other health professionals in that psychoeducational and cognitive-behavioral interventions focusing on sleep hygiene and sleep parameters can improve global sleep and consequently may benefit cognitive functioning.
